# Fleas as parasites of the family Canidae

**DOI:** 10.1186/1756-3305-4-139

**Published:** 2011-07-18

**Authors:** Gerhard Dobler, Martin Pfeffer

**Affiliations:** 1 Bundeswehr Institute of Microbiology, Department of Virology and Rickettsiology, Neuherbergstrasse 11, D-80937 Munich, Germany; 2 Institute of Animal Hygiene and Public Veterinary Medicine, University of Leipzig, An den Tierkliniken 1, D-04103 Leipzig, Germany

## Abstract

Historically, flea-borne diseases are among the most important medical diseases of humans. Plague and murine typhus are known for centuries while the last years brought some new flea-transmitted pathogens, like *R. felis *and *Bartonella henselae*. Dogs may play an essential or an accidental role in the natural transmission cycle of flea-borne pathogens. They support the growth of some of the pathogens or they serve as transport vehicles for infected fleas between their natural reservoirs and humans. More than 15 different flea species have been described in domestic dogs thus far. Several other species have been found to be associated with wild canids. Fleas found on dogs originate from rodents, birds, insectivores and from other Carnivora. Dogs therefore may serve as ideal bridging hosts for the introduction of flea-borne diseases from nature to home. In addition to their role as ectoparasites they cause nuisance for humans and animals and may be the cause for severe allergic reactions.

## Background

Fleas (Order Siphonaptera) form a unique group of insects. They have evolved in the early Cretaceous or Jurassic ages between 125 to 150 million years ago, probably together with the evolution of marsupials and insectivors. Historically, fleas are among the most important ectoparasites of humans in that several species are the natural vectors of several important infectious diseases, like plague. Today, some 15 families with a total of about 220 genera and some 2,500 species of fleas can be distinguished [1].Out of these, five families and 25 genera are ectoparasites of birds, and all other fleas parasitize mammals. Most fleas of veterinary importance are grouped in the families Pulicidae, Ceratophyllidae, Leptopsyllidae and Vermipsyllidae. Rarely, members of other families (Hystrichopsyllidae, Rhopalopsyllidae) may be found on domestic animals. Besides domestic cats, dogs may play a peculiar role as bridging hosts for fleas of different wild animals, domestic animals and humans, as they will come into contact with different animals during their seeking behaviour and therefore acquire the fleas of different animals. Fleas may play different roles as parasites in mammals. They may act as vectors to transmit pathogens. They may play a role as intermediate hosts of parasites and can be an ectoparasitic nuisance in animals and humans which may cause allergic reactions.

## Review

### Morphology and Development

Fleas are wingless, 1-8 mm in size with laterally compressed bodies composed of a blunt head, a compact thorax and a fairly large, rounded abdomen. The colour is usually dark brown. Small eyes may or may not be present. The bodies of adult fleas bear many spines which are used for species determination. Among them are the combs or ctenidia, a row of enlarged sclerotized spines on the head (genal ctenidium), which are always absent in fleas parasitizing birds, or on the prothorax (pronotal ctenidium) which may be present or absent.

**Figure 1:**
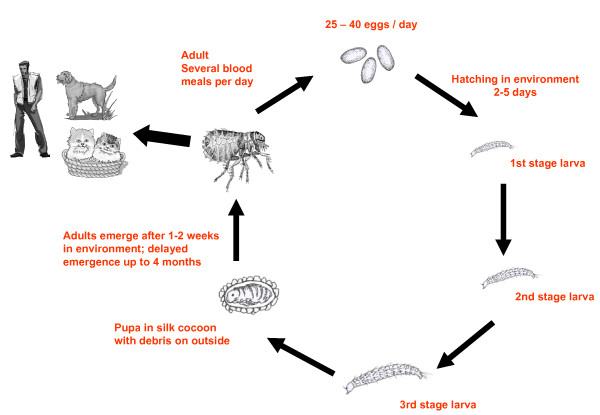
**Schematic Life cycle of *Ctenocephalides felis***.

Fleas develop from eggs to larvae (usually consisting of three instar generations) and pupae into the adult stage (Figure 1). Females lay up to 25 eggs per day and a total of several hundreds during their life. The speed of development is dependent on environmental conditions. Higher temperatures may increase the generation time of fleas. Colder temperatures and higher humidity influence the longevity of fleas in the absence of available hosts. Fleas of domestic animals oviposit their eggs randomly into the environment. The developing larvae feed on organic matter. In some flea species, like *Ctenocephalides (C.) felis*, the dried, blood-rich feces of adult fleas will be used for food by the larvae. Others may feed on small arthropods, if available in the surroundings or even use other larvae as food (cannibalism). The development through the three instar larval stages lasts about 2 to 3 weeks. The third instar larval stage spins a silk cocoon including dust, debris and grains of sand, which are bound together by silk and which act as camouflage for the pupa during development to adult stages. The adult flea emerges almost colourless, but melanisation starts immediately so that imagos darken within a short period of time. Adult fleas feed frequently several times during the day, which is necessary as most of the blood is excreted in a semi-digested stage and these flea feces may be frequently used as food by the larvae.

### Fleas of domestic dogs

Fleas have a global distribution. As far as data are known from different countries worldwide, fleas are also found in dogs all over the world. To our best knowledge, no comprehensive review of the occurrence and frequency of fleas in dog populations is available. We therefore evaluated the published literature of the years 1980 to 2010 for occurance and frequency of fleas in the dog populations of the different countries (Table 1). This literature search provided evidence of at least 15 different species of fleas found on domestic dogs. The data show that the most prevalent flea species found globally in domestic dogs is the cat flea (*C. felis*, Figure 1). Prevalence rates range form 5% to 100%. The dog flea (*C. canis*) also occurs globally, but in lower rates than the cat flea. The presence of the human flea, *Pulex (P.) irritans*, in many countries and on average on up to 10% of tested dogs may show evidence of the close contact between humans and dogs. Several other species of fleas, originally feeding on birds (hen fleas), rodents (rat fleas) or insectivores (hedgehog fleas) are found on domestic dogs which indicates that dogs due to their habits will come into contact with other domestic and wild animals and pick up fleas of these animals. This contact with other animals, together with the close contacts with humans on the other hand, predestine dogs as a bridging vector for ectoparasites from domestic or wild animals to humans and for the agents which they might transmit while blood-feeding.

**Table 1:** Prevalence of flea infestation in dog populations of various countries (according to different references) (*A.: Archaeopsylla; C.: Ctenocephalides; Ca.: Cediopsylla; Cr.: Ceratopsyllus; Ct.: Ceratophyllus; E.: Echidnophaga; M.: Megabothris; P.: Pulex; Pa. Paraceras; R.: Rhopalopsyllus; T.: Tunga; X.: Xenopsylla; *n.d. no data provided)

Country	No. of dogs tested	Infestation rate (% of tested dogs carrying fleas)	Flea species detected	Literature
Albania	181	76%	*C. canis*	[76]
			
		5%	*C. felis*	
			
		8%	*P. irritans*	

Argentina	116	98%	*C. canis*	[77]

Australia	116	85%	*C. felis*	[78]
			
		6%	*C. canis*	
			
		13%	*E. gallinacea*	

Austria	129	81%	*C. felis*	[65]
			
		19%	*C. canis*	
			
		7%	*A. erinacei*	

Brazil	61	69%	*T. penetrans*	[79]

Brazil	101	64%	*C. felis*	[80]
			
		2%	*Rh. lutzi*	
			
		2%	*T. penetrans*	

Brazil	41	44%	*C. felis*	[81]

Brazil	46	39%	*C. canis*	[82]
			
		17%	*C. felis*	

Chile	3000	42%	*C. felis*	[83]
			
		39%	*C. canis*	
			
		19%	*P. irritans*	

Denmark	140	54%	*C. felis*	[84]
			
		42%	*C. canis*	

France	392	89%	*C. felis*	[85]
			
		10%	*C. canis*	
			
		1.3%	*A. erinaceus*	
			
		0.8%	*P. irritans*	

Germany	1922	4%	*C. felis*	[73]
			
		0,9%	*C. canis*	
			
		< 0,1%	*A. erinacei*	
			
		< 0,1%	*P. irritans*	
			
		< 0,1%	*Ce. gallinae*	
			
		< 0,1%	*Cr. garei*	
			
		< 0,1%	*Pa. melis*	
			
		< 0,1%	*M. sp*.	

Germany	48	46%	*C. felis*	[86]
			
		44%	*C. canis*	
			
		19%	*A. erinacei*	
			
		10%	*P. irritans*	

Germany	163	28%	*C. felis*	[87]
			
		14%	*C. canis*	
			
		58%	*A. erinacei*	

Greece	129	71%	*C. canis*	[74]
			
		40%	*C. felis*	
			
		1%	*P. irritans*	
			
		1%	*X. cheopis*	

Hungary	2267	8%	*C. canis*	[72]
			
		6%	*C. Felis*	
			
		< 0,1%	*P. Irritans*	

Iran	756	8%	*C. felis*	[88]
			
		1%	*C. Canis*	
			
		0,9%	*P. irritans*	
			
		0,5%	*X. cheopis*	
			
		0,3%	*Ca. simplex*	

Ireland	103	75%	*C. Felis*	[89]
			
		18%	*C. canis*	
			
		4%	*A. erinacei*	
			
		2%	*Ca. spp*.	

Italy	1376	16%	*C. felis*	[90]
			
		2%	*C. canis*	

Korea, Republic of	103	75	*C. canis*	[91]

Laos	3	n.d.	*C. felis*	[92]
			
		n.d.	*C. canis*	
			
		n.d.	*C. orientis*	

Mexico	1803	25%	*C. felis*	[93]
			
		5%	*C. canis*	

New Caledonia	8	100%	*C. felis*	[38]

Nigeria	396	33%	*C. canis*	[94]
			
		7%	*P. irritans*	
			
		0,5%	*T. penetrans*	

Pakistan	n.d.	34%	*C. felis*	[95]

Poland	50	100%	*C. canis*	[96]

Spain	109	100%	*P. irritans*	[97]

	87	71%	*C. felis*	[98]
			
		13%	*C. canis*	
			
		16%	*P. irritans*	

Spain	744	95%	*C. felis*	[99]
			
		10%	*C. canis*	
			
		1%	*P. irritans*	
			
		0,1%	*E. gallinacea*	

United Kingdom	60	78%	*C. felis*	[75]
			
		20%	*C. canis*	
			
		2%	*A. erinacei*	

United Kingdom	2653	12%	*C. felis*	[100]
			
		< 0,1%	*C. canis*	
			
		< 0,1%	*A. erinacei*	
			
		< 0,1%	*P. irritans*	
			
		< 0,1%	*Ca. fasciatus*	

USA	11	61%	*C. felis*	[101]
			
		21%	*C. canis*	

USA	100	8%	*P. irritans*	[102]

Since the 1990s, many powerful anti-ectoparasitics for domestic animals were commercialized which can be easily applied by the animal owners. The use of these repellents may have changed the prevalence of flea infestation of domestic dogs, especially in developed countries where these substances are in use. Unfortunately, studies showing the effect on the prevalence of flea infestation in domestic dog populations have never been conducted. However, in most of the studies cited above, dogs from countries were tested, where repellents are not in use for dogs. Whether in the studies cited from industrialized countries the effect of the use of ectoparasitics since the 1990s had an effect on the rate of flea infestation or whether other factors like improved animal hygiene may be also involved remains speculative.

### Fleas of wild canids

The family Canidae contains the two tribes Vulpini and Canini with a total of 13 genera and 35 species. However, so far, only few studies have been conducted to examine the flea infestation of wild canids and thus, only limited data are available on the prevalence of fleas and on the species composition of fleas in wild canids. The results of an extended literature search on all 35 canid species yielded only data on flea infestations for six different species (Table 2). Most data are available on fleas on red foxes (*Vulpes (V.) vulpes*). An Austrian report reviews several studies done in the federal states of Burgenland, Lower Austria and Steiermark in Austria [2]. This review summarized five different studies where fleas were collected from red foxes [3-7]. A total of 13 different flea species were collected from foxes in Austria. The most prevalent species was *Chaetopsylla (Ch.) globiceps *which was found from 10 to more than 30% of the foxes tested. Interestingly, the second most prevalent flea species in two of the studies reviewed was the human flea, *P. irritans*. Furthermore, the cat flea and the dog flea were among others found on wild mammals (badger, squirrels, rats, rabbits, chicken, hedgehog). This diverse flea fauna of foxes in Austria may indicate that the red fox may come into contact with different wild and domestic animals potentially used as prey (rodents, rabbits, chicken) or as co-habitation (badger).

**Table 2:** Available data on the prevalence rates of flea infestation in different species of the family Canidae in different countries (*A.: Archaepsylla; C.: Ctenocephalides; Ce.: Cediopsylla; Ch.: Chaetopsylla; D.: Dactylopsyllus; E.: Euhoplopsyllus; O.: Oropsylla; Or.: Orchopeas; P.: Pulex; Pa.: Paraceras; R.: Rhopalopsyllus; X. Xenopsylla*).

Canid species	Country	Flea prevalence	Flea species detected	Literature
*V. vulpes*	Austria	10-30%	*Ch.globiceps*	[3-7]
			
		3-13%	*Ch. trichosa*	
			
		0.5-20%	*P. irritans*	
			
		1-2%	*C. felis*	
			
		1-7%	*C. canis*	
	
	Spain	100%	*P. irritans*	[98]
	
	Hungary	43%	*P. irritans*	[103]
	
		37%	*Ch. globiceps*	
			
		12%	*Ch. trichosa*	
			
		4%	*Pa. melis*	
			
		11%	*C. canis*	
			
		3%	*A. erinacei*	

*V. velox*	U.S.A.	100%	*P. irritans*	[9,10]
			
		92%	*P. simulans*	
			
		3%	*E. glacialis*	
			
		<1%	*O. hirsuta*	
			
		<1%	*E. affinis*	
			
		<1%	*D. percernis*	

*Urocyon cineroargenteus*	U.S.A.	38%	*P. simulans*	[8-10,12]
			
		<1%	*C. felis*	
			
		<1%	*C. canis*	
			
		<1%	*Ce. inequalis interrupta*	
			
		<1%	*Or. laens*	

*Urocyon littoralis*	U.S.A.	98%	*P. irritans*	[13]

*Cerdocyon thous *	Brazil	89%	*Rh. lutzi*	[14]
			
		7%	*P. irritans*	
			
		2%	*C. canis*	
			
		1%	*C. felis*	
			
		2%	*X cheopis*	
	
	Bolivia	0%	*none*	[104]

*Lycalopex gymnocercus*	Bolivia	n.d.	*P. irritans*	[104]

A study on the role of Swift foxes (*V. velox*) for the transmission of plague between prairie dogs in Texas, found that the main flea ectoparasites in these dog population was the human flea (*P. irritans*) [8]. In six out of ten foxes *P. simulans *was also detected. In one out of ten foxes *Oropsylla (O.) hirsuta*, a flea of prairie dogs was detected. These data confirmed earlier works of other groups that *P. irritans *is the most prevalent flea species in Swift foxes[9,10]. Two other flea species, *Euhoplopsyllus affinis *and *Dactylopsylla percernis *were detected also on foxes in Texas [11]. In a study in Californian gray foxes (*Urocyon cinereoargentus*) 22/54 foxes (42%) were found to be infested by fleas [12]. Five different species of fleas were identified. More than 90% of the fleas found were identified as *P. simulans*. 3% were classified as *C. felis*, one percent each as *C. canis*, *Cediopsylla (Ca.) inequalis interrupta *and *Orchopeas (Or.) laens*. A study in a closely related fox species, the island fox (*Urocyon littoralis*) on the California channel island Santa Cruz, showed that 98% of all foxes tested were infested by one single flea species, *P. irritans *[13]. In Spain, results of a survey of fleas in carnivorous mammals showed that only *P. irritans *was found on red foxes (*V. vulpes*). During a leishmaniosis study in the Brazilian State of Bahia, crab-eating foxes (*Cerdocyon thous*) were studied [14]. All 18 specimen tested were infested with fleas. Five species of fleas were identified. The most frequent species found was *Rhopalopsyllus (Rh.) lutzi *(89%), *P. irritans *(7%), *C. canis *(2%) *C. felis *(1%) and *Xenopsylla (X.) cheopis *(2%). Probably, fleas parasitizing foxes and other canids may also parasitize domestic dogs in where there is adequate contact between dogs and wild foxes and canids. The summarized data enlarge the number of potential parasitizing flea species on domestic dogs to more than 30 different species.

### Fleas as vectors of pathogens

Beside their role as ectoparasites, the major medical importance of fleas is their role as vectors of various pathogens to humans and animals. While more than 550 arboviruses are found in arthropods, so far surprisingly, no arbovirus has been detected which uses fleas as a biological vector. However, there is some evidence that some viruses, namely feline leukemia virus and myxomatosis virus, under artificial laboratory conditions could be mechanically transmitted by fleas [15,16]. The importance of this observation for the natural transmission of these viruses remains to be elucidated.

#### Plague

Fleas are mainly the vectors of bacteria. Historically, the most important bacterial agent which is transmitted by fleas is the plague bacterium, *Yersinia (Y.) pestis*. Human disease caused by *Y. pestis *has been historically associated with rats, mainly with *Rattus (Ra.) norvegicus *and *Ra. rattus*. The origin of *Y. pestis *is now thought to be the steppe regions of Central Asia. In nature, the plague agent is transmitted by fleas among the rodent populations. Mainly rodent fleas are involved in the natural transmission, and more than 80 flea species belonging to different genera were found to transmit *Y. pestis *in nature [17,18]. These flea species mainly parasitize rodents, but they may be occasionally found on pets and also on dogs which can be shown by the occasional detection of fleas of wild rodents or other wild animals on dogs. The transmission of plague to humans via flea bites is almost exclusively through the oriental rat flea (*X. cheopis*) which played the major role as bridging vector between the rodent and rat populations and humans.

While the role of cats for the transmission of plague has been established for a long time [19], dogs are considered to be less susceptible to plague and their role for the transmission of *Y. pestis *has not been established equally well. Dogs seem to develop some clinical illness after infection with *Y. pestis*, including symptoms like fever, lethargy and bubos [20]. Epidemiological data show that plague patients are significantly more likely reported for sleeping in the same bed as a dog [21]. These data indicate that probably a long and close contact with dogs and their fleas is necessary for the transmission from dogs to humans [22]. These epidemiological data are strengthened by experimental data, showing that the flea species found most commonly on dogs, the cat flea and the dog flea, are considered to be poor vectors of *Y. pestis *due to their poor ability to become blocked [23]. They may possess a small although not essential importance by their ability to become pestiferous. The mouth parts may be contaminated with *Y. pestis *during blood sucking of bacteriaemic blood. The transmission may occur mechanically or by a newly discovered unblocked transmission mechanism recently shown to play a role in the transmission of plague in the flea species *O. montana *[24]. However, a role of *P. irritans*, which is found frequently in low prevalence rates on dogs (see above), is under discussion and has not yet been resolved [25]. The role of another flea, occassionally detected on dogs, *Echidnophaga (E.) gallinacea*, in the transmission of plague is unclear, although it is known to become infected with *Y. pestis *[26]. However, it is also thought to be a poor vector for the agent due to its stick-tight behaviour [23,26]. The role of *Tunga (T.) penetrans *as a plague vector is unknown [25]. Possibly the male fleas, which are free hematophagous ectoparasites, may act as pestiferous vectors while the females of *T. penetrans *are not capable to do so, because they are embedded in the host epidermis [27]. No data are available on *Archeopsylla erinacei *and its potential role as a vector of *Y. pestis*. The ability of *Rh. lutzi *to transmit the plague bacterium is unknown. However, a non-identified *Rh. sp*. is listed as vector in an established wild rodent plague focus in South America [28]. Fleas of the genus *Megabothris *were also involved in the natural transmission of *Y. pestis *in natural foci in the northwestern U.S.A. [28], while information on species of the genera *Ceratopsyllus*, *Paraceras*, *Cediopsylla *and *Ceratophyllus *to our best knowledge is not available.

#### Rickettsioses

There are mainly two species of rickettsiae which are naturally transmitted by fleas, *Rickettsia (R.) typhi*, the pathogen of murine typhus and *R. felis*, a recently discovered *Rickettsia *species causing flea-borne spotted fever.

Murine typhus is a zoonosis which is maintained in nature mainly by a flea-rat-flea transmission cycle [29]. Murine typhus is one of the few rickettsioses which are distributed globally except in the Antarctic [29]. Rats of the genus *Rattus*, mainly *R. rattus *and *R. norvegicus *are of major importance for the primary transmission cycle as vertebrate hosts. Besides these two species, many other wild and domestic animals may serve as additional vertebrate hosts. Among them, dogs were also found to become infected with *R. typhi *in different areas of the world. In Spain, between 10 to 12% of dogs showed antibodies against *R. typhi*, while during an outbreak of murine typhus in Austin, Texas, U.S.A., 44% of dogs tested exhibited antibodies agains *R. typhi *[30-33]. Because of these data, the potential role of pet and stray dogs during urban outbreaks of murine typhus has to be readdressed.

*R. typhi *has deen detected in at least ten species of fleas of the genera *Ctenocephalides*., *Echidnophaga*, *Leptopsylla*, *Monopsyllus*, *Nosopsylla*, *Pulex *and *Xenopsylla *[29]. Among them is *C. felis *which is frequently found on dogs all over the world and the most prevalent flea species on dogs in many areas (Table 1). Several other flea species are known to play a role as actual or potential vectors of *R. typhi*, among them are *E. gallinacea*, *P. irritans *and *X. cheopis*. They are found occasionally on dogs and therefore may support an urban dog-flea-dog transmission cycle (Table 1). *X. astia*, *X. bantorum*, *X. brasiliensis*, *Leptopsylla (L.) segnis *and *Nosopsylla (N.) fasciatus *are primary rodent fleas which may accidentally feed on humans or on dogs [1].

*R. felis *is the pathogen of flea-borne spotted fever. Interestingly, together with *R. typhi*, it is the only other *Rickettsia *species which seems to be globally distributed. Both of these rickettsiae are transmitted by fleas. While the agent of murine typhus has been known for many decades, the agent of flea-borne spotted fever was only detected recently. In 1990, a rickettsia-like organism in an adult flea colony in the Elward Laboratory, Maryland was detected and named "ELB agent" [34]. Later, the further characterization of this agent resulted in the classification as a spotted fever group rickettsiae [35]. In recent years, the widespread geographical distribution of *R. felis *and of the disease caused by this agent increased, indicating that *R. felis *is the most widely distributed *Rickettsia *species.

The cat flea (*C. felis*) was identified as the primary vector and reservoir of *R. felis*. So far, this *Rickettsia *species has been detected in more than 30 countries on five continents except Antarctica [36-41]. The reason for this almost universal distribution may be seen in the global distribution of *C. felis *which finally transported its ectoparasites and their endoparasites into all parts of the world. The high infection rates of colonized cat flea populations from 40 up to 90% [42], together with experimental data, imply that *R. felis *is well-adapted to its vector including transstadial and transovarial transmission leading to the high infection rates of fleas [43,44]. Although the cat flea is thought to be most important as a vector, *R. felis *was detected in at least eleven other flea species [36]. Among them are *P. irritans*, *Archaeopsylla (A.) erinacei*, *E. gallinacea*, *C. canis*, *T. penetrans *and *X. cheopis*. These species are found either sporadically or more frequently also on dogs.

Although a number of serological studies have been conducted to identify the mammalian host(s) of *R. felis*, no definitive mammalian host has been identified so far. Among the peri-domestic mammals which exhibit antibodies against *R. felis*, cats, dogs and opossums are found [36]. Cats are believed to be the most important hosts for the cat flea and for the rickettsiae transmitted by cat fleas. In different areas of the world antibody seroprevalence rates of 4 to 100% of cats were reported in several studies (summarized in [36]). However, the definitive role of the cat as a mammalian host supporting the life cycle of *R. felis*, has not been established. While the transmission of fleas to cats had been proven either by serology or by molecular biology [44], the horizontal transmission of *R. felis *from mammals to fleas so far has not been shown [45]. Therefore, the role of cats for the life cycle of *R. felis *remains unclear. Even more unclear is the role of domestic dogs for the maintainance of the natural life cycle of *R. felis*. Only few seroprevalence studies in dogs are available. In Spain, 16% of dogs showed specific antibodies against *R. felis *[31]. Two other reports indicate an association of PCR-positive dogs to the occurance of sporadic human cases of flea-borne spotted fever. In Germany and in Spain, *R. felis*-infected dogs were present in families with human cases [46,47]. The dog seems to play the role as a transport vehicle for *C. felis *and also for its parasite, *R. felis *to humans. So far, no wild animal reservoir of *R. felis *has been detected. There is, however, some evidence, that the opossum (*Didelphis virginiana*) may play a role [48,49].

Another *Rickettsia *species was recently found in fleas. In the USA, a sylvatic transmission cycle of *R. prowazekii *was detected [50]. When exploring this transmission cycle, *R. prowazekii *could be detected in lice and in squirrel fleas (*Or. howardi*). The exact transmission between the squirrels and from squirrels to humans remains unclear. However, it is hypothesized that *R. prowazekii *is excreted by the fleas (and lice) with their feces. After drying of feces, the pathogen may be inhaled as aerosol and then may cause typhus. A role for dogs within this so-called sylvatic transmission has not been postulated so far. However, dogs may serve as mechanical carriers of fleas from outside to the home and therefore serve as a bridging host.

#### Bartonellosis

The third group of bacterial pathogens transmitted by fleas are members of the genus *Bartonella*. Among the more than 20 species of *Bartonella*, at least eleven species are known to cause human disease [51,52]. *Bartonella (B.) henselae *and *B. clarridgeiae *are transmitted by fleas and they cause a disease in humans, cat-scratch disease. Dogs may be either the primary reservoir or the accidental hosts for at least seven *Bartonella *species (*B. vinsonii subsp. berghoffii, B. quintana, B. henselae, B. clarridgeiae, B. washoensis, B. elizabethae, B. koehlerae*) [53]. Epidemiological data, however, indicate that dogs are accidental hosts rather than reservoir hosts [51]. *B. henselae *and *B. clarridgeiae *are the two species which are mainly detected in fleas from dogs. They so far have been detected in four continents (Table 3).

**Table 3:** Detection of Bartonella spp. in fleas collected from dogs from different countries (*A. Archaeopsylla; C.: Ctenocephalides; Ce.: Ceratopsyllus*)

Country	Dogs tested	Flea species tested for Bartonella	No of fleas tested	No of fleas positive for Bartonella (B.) species:	Literature
France	84	*C. felis, C. canis, P. irritans*	317	*B. henselae *2 *B. clarridgeiae *12	[53]

Germany	49	*C. felis *	114	*B. henselae *0 *B. clarridgeiae *0	[53]
			
		*C. canis *	4	*B. henselae *0 *B. clarridgeiae *0	
			
		*A. erinacei*	26	*B. henselae *0 *B. clarridgeiae *0	
			
		*Ce. gallinae*	2	*B. henselae *0 *B. clarridgeiae *0	

Laos	3	*C. felis *	23	*B. clarridgeiae *1	[39]

Lebanon	2	*C. canis *	50	*B. henselae *0 *B. clarridgeiae *0	[37]

New Caledonia	8	*C. felis *	20	*B. clarridgeiae *1	[38]

United Kingdom	31	*C. felis *	280	*B. henselae *0	[16]

Besides these two pathogens of the genus *Bartonella*, dogs and canids seem to play a role as putative natural hosts for *B. vinsonii subsp. berkhoffii *[54-56]. Various data show that stray dogs in the tropics exhibit high antibody prevalence rates against *B. vinsonii subsp. berkhoffii*. In sub-Saharan Africa and in Asia 26% to 65% of domestic dogs tested were antibody positive against this species [57,58]. In contrast, dogs in non-tropical regions (Europa, U.S.A., northern Africa) were seropositive only in < 5% [52,59,60]. However, in California, *B. vinsonii subsp. berkhoffii *was detected by serology in up to 35% and in 28% by PCR in coyotes (*Canis latrans*). This high prevalence and the apparent chronic bacteriaemia imply a role of the coyote as natural host for *B. vinsonii subsp. berkhoffii *[59]. Possibly, the frequent contacts of stray dogs with wild canids cause the high prevalence rates while the contacts of domestic dogs with coyotes in California seem to be limited. *B. vinsonii subsp. berkhoffii *has also been incriminated as a cause of human heart disease [61]. The infection of humans by flea-borne bartonellae mainly occurs via cat scratches. An infection of cats via infected fleas could be experimentally demonstrated, although this way is thought not to play a major role in the transmission and epidemiology of flea-borne *Bartonella*. The infection of humans via fleas seems possible, however this mode of infection also does not seem to play a major role for the epidemiology of *Bartonella*. Sporadic cases of transmission of bartonellae by dog scratches or dog bites document the potential risk of transmission from dog to human and therefore may also involve dogs as bridging hosts from animals to humans [62,63].

#### Miscellaneous

A number of other bacterial pathogens have been isolated from fleas. These are *Coxiella burnetii, Francisella tularensis, Staphylococcus aureus, Salmonella enteritidis, Borrelia burgdorferi, Borrelia duttoni, Listeria monocytogenes, Y. pseudotuberculosis, Erysipelothrix rhusiopathiae, Burkholderia (Bu.) mallei, Bu. pseudomallei*, and *Brucella abortus *[1]. Probably, most of these pathogens were detected by chance after a bacteriaemic bloodmeal. The potential to serve as vectors for the agents listed, although unknown and never tested experimentally, seems to be minimal.

### Fleas as intermediate hosts of parasites

Fleas play an important role as intermittent hosts in the development of at least three species of tapeworms. The double-pored tapeworm (*Dipylidium caninum*) is dependent on fleas as intermediate hosts and on dogs as final hosts. It is the most prevalent tapeworm in dogs and occurs world-wide. Prevalence data are not available for most parts of the world. In central Europe the carrier rate of dogs ranges from 0.5 to 6% in Austria and Poland to more than 65% in Albania [64-67]. In Mexico, the prevalence rate reached almost 50% [68]. The adult tapeworms in the small intestine produce proglottids which are passed in the feces. After desiccation in air, the eggs are expelled form the proglottids and then they can be digested by flea larvae due to their chewing mandibles, but not by adult fleas. The eggs develop rapidly into flea pupae. The development is completed if the adult flea is accidentally ingested by humans or by dogs and cats. There, the adult worm is liberated from the cysticercoid and attaches to the gut of the new host. The cat flea (*C. felis*), the dog flea (*C. canis*) and the human flea (*P. irritans*) play a major role in the life cycle of this tapeworm. In Africa, the warthog flea (*E. larina*) was found to be responsible in some cases for the infection of domestic dogs [1]. Mainly children are at risk when playing with infected pets due to their close contacts with the animals and the low personal hygiene standards. Two other tapeworms, the dwarf tapeworm (*Rodentolepis nana*, syn. *Hymenolepis (H.) nana*) and the rodent tapeworm (*H. diminuta*) use fleas and other insects as intermediate hosts for their development from eggs to cysticercoids. For these worms, humans may play a role as accidental final hosts. Humans are infected by incidental ingestion of infected rat fleas (*X. cheopis*, *N. fasciatus*) and they may play a role as accidental final hosts. One other worm, the microfilaria *Acanthocheilonema (Dipetalonema) reconditum *is known to be transmitted by cat fleas to humans and to dogs [69]. Dogs and humans seem to form aberrant hosts for this microfilaria species which may cause severe eye disease and inflammatory skin disease also [69].

### Fleas as ectoparasites of dogs

Several species of fleas may pose a threat as ectoparasites for humans and pet animals. From a total of ten species of fleas of the genus *Tunga*, *T. penetrans *is the only species which may be found on dogs and humans. A second species, *T. triamillata*, may be found on humans, but so far has not been detected on dogs [70]. *T. penetrans *is known to occur mainly in southern and Central America and in Africa. The females of the sand flea penetrate the skin of the host to the basal layer of the corium [71]. There, they feed on blood and tissue exsudates produced by the host's inflammatory response. Only female fleas cause tungiasis. The infestation of the skin may cause severe damage by inflammatory response or by bacterial superinfection. Dogs are commonly infested and especially the snout and the pads of the feet may be involved. Infestations of humans or animals occurs if adult females which developed in the soil from larvae to pupae and to imagos which then may come into contact with the skin of suitable vertebrate hosts. Penetration of the skin occurs within minutes. Within 7 to 14 days, the females increase in size up to 10 mm and they produce up to 200 eggs which are expelled. If conditions are favourable, larvae will hatch and develop to pupae and imagos again. In tungiasis, dogs may play a role as vertebrate hosts of the flea and as a bridging host, importing the fleas into the surroundings of houses or into houses where they may complete their life cycle and subsequently infest humans.

Although not in the scope of this review, which is concentrating mainly on the role of dogs and wild canids as hosts for fleas and flea-transmitted diseases, the importance of domestic cats for the maintenance and transmission of fleas and flea-borne diseases should be mentioned. Several studies in different countries testing for or comparing the infestion of fleas on dogs and cats clearly show that domestic cats have similar or higher infestation rates with fleas than dogs [41,72-75]. These limited studies also underscore the great importance of cats as animal bridging hosts for the transmission of fleas (mainly *C. felis*) to humans.

## Conclusions

Dogs and their ectoparasites of the order Siphonaptera play major, but different roles as vectors or hosts of pathogens. Among the diseases transmitted by fleas, the historically most important disease of mankind, plague, is of special importance. Among the rickettsioses transmitted by fleas, one human disease, murine typhus, was characterized in the early 1900s while flea-borne spotted fever is a disease which has been recognized only since 1990. Flea-transmitted *Rickettsia *species are the only rickettsiae which are globally distributed. Fleas may directly ingest the pathogens into the host during their blood meals or pathogens are excreted with the fleas' feces and will then be inoculated by scratching. Also, direct injection during the blood-feeding of flees seems possible. More recently, the role of fleas and dogs for the epidemiology and epizootology of several *Bartonella *species was detected. The role of fleas as intermediate hosts for the developmental cycle of tapeworms is recognized and may be of medical importance in situations with low personal hygiene standards. Finally, fleas occur as ectoparasites on dogs and also on humans, and tungiasis may cause severe skin infections. In the future more pathogens may be detected which are in some way of transmission or development associated with fleas.

## Competing interests

Both authors declare that they do not have any financial competing interests (political, personal, religious, ideological, academic, intellectual, commercial or any other) in relation to this manuscript.

## Authors' contributions

Both authors contributed equally to this publication and have approved the final version of the manuscript.
